# Supplementary material for: Structure-guided functional studies of plasmid-encoded dihydrofolate reductases reveal a common mechanism of trimethoprim resistance in Gram-negative pathogens
Source: Commun Biol. 2022 May 13;5:459. doi: 10.1038/s42003-022-03384-y (PMC9106665; doi:10.1038/s42003-022-03384-y)
Supplement: Supplementary file 2 — Supplemental Information [file 42003_2022_3384_MOESM2_ESM.pdf]

Structure-guided functional studies of plasmid-encoded dihydrofolate reductases reveal a common mechanism of trimethoprim resistance in Gram-negative pathogens

Jolanta Krucinska[1], Michael N Lombardo[1], Heidi Erlandsen[2], Alexavier Estrada[1], Debjani Si[1], Kishore Viswanathan [1], Dennis L Wright[1]*

[1] Department of Pharmaceutical Sciences, University of Connecticut, 69 N. Eagleville Rd., Storrs, Connecticut 06269, United States

[2] Center for Open Research Resources & Equipment (COR^{2}E), University of Connecticut, 91 N. Eagleville Rd., Storrs, Connecticut 06269, United States

a

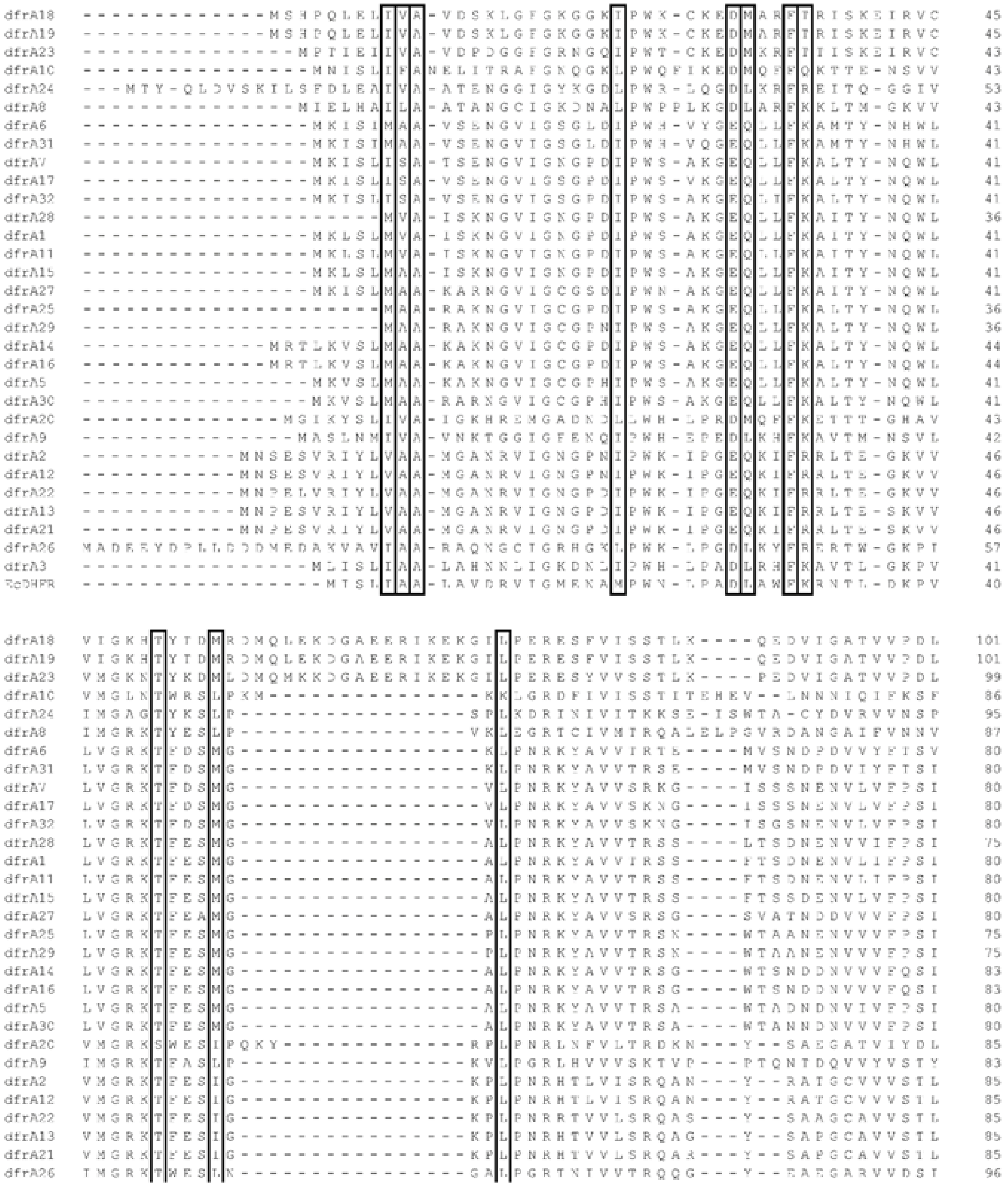

```
dfrA3    VMGRRTFESIG----------------RPLPGRRNVVVSRNPQ---W--QAEGVEVAPSL   80
EcDHFR   IMGRHTWESIG----------------RPLPGRKNIILSSQPG---T--DD-RVTWVKSV   78

dfrA18   RAVINL-----Y-----ENTDQRIAVIGGEKLYIQALSS--ATKLHMTIIPREFD--CDR   147
dfrA19   RAVINL-----Y-----ENTDQRIAVIGGEKLYIQALSS--ATKLHMTIIPREFD--CDR   147
dfrA23   RAVLNR-----Y-----HDSDQRIAVIGGEKLYVQALAS--AKKVHLTVMPRSYK--CDR   145
dfrA10   ESFLEA-----F-----RDTTKPINVIGGVGLLSEAIEH--ASTVYMSSIHMVKPVHADV   134
dfrA24   EDALRMVGRIIDEKEEQGRDRPRVFVIGGASIYQALMPF--VSTLHWTEVHVEQL-PEEI   152
dfrA8    SDAMRFAQ---E-----ESVGDVAYVIGGAEIFKRLALM--ITQIELTFVKRLYE--GDT   135
dfrA6    ESALAY-----L-----DHTTTHVFVSGGGEIYKALIEQ--ADVIHLSVIHKHTS--GDV   126
dfrA31   ESALSY-----L-----DNTTTHVFVSGGGEIYKALIEQ--ADVIHLSVIHKHIS--GDV   126
dfrA7    EIALQE-----L-----SKIIDHLYVSGGGQIYNSLIEK--ADIIHLSTVHVEVE--GDI   126
dfrA17   ENALKE-----L-----SKVTDHVYVSGGGQIYNSLIEK--ADIIHLSTVHVEVE--GDI   126
dfrA32   ENALQE-----L-----SKITDHVYISGGGQIYESLIEK--ADIIHLSTIHVEVE--GDI   126
dfrA28   KDALTN-----L-----KKITDHVIVSGGGEIYKSPIDQ--VDTLHISTIDIEPE--GDV   121
dfrA1    KDALTN-----L-----KKITDHVIVSGGGEIYKSLIDQ--VDTLHISTIDIEPE--GDV   126
dfrA11   KDALTN-----L-----KKITDHVIVSGGGEIYKSLIDQ--VDTLHISTIDIEPE--GDV   126
dfrA15   DEALNH-----L-----KTIIDHVIVSGGGEIYKSLIDK--VDTLHISTIDIEPE--GDV   126
dfrA27   EAAMRE-----L-----KTLTNHVVVSGGGEIYKSLIAH--ADTLHISTIDSEPE--GNV   126
dfrA25   DEAMGR-----L-----GEITDHVIVAGGGEIYHETIPM--ASTLHVSTIDVEPE--GDV   121
dfrA29   DEAMGR-----L-----GEITDHVIVAGGGEIYHETIPM--ASTLHVSTIDVEPE--GDV   121
dfrA14   EEAMDR-----L-----AEFTGHVIVSGGGEIYRETLPM--ASTLHLSTIDIEPE--GDV   129
dfrA16   EEAMDR-----L-----AEFTGHVIVSGGGEIYRETLPM--ASTLHLSTIDIEPE--GDV   129
dfrA5    EEAMYG-----L-----AELTDHVIVSGGGEIYRETLPM--ASTLHISTIDIEPE--GDV   126
dfrA30   EEAMGG-----L-----AKLNGHVIVSGGGEIYRETLPM--ASTLHVSTIDIEPE--GDV   126
dfrA20   KEVAQH-----L-----EGKNLTCFITGGAQIYQLALETGLLNEMYVTQVHNTFE-EADT   134
dfrA9    QIAVRTASLLVD-----KPEYSQIFVIGGKSAYENLAAY--VDKLYLTRVQLNTQ--QDT   134
dfrA2    SHAIALA----------SELGNELYVAGGAEIYTLALPH--AHGVFLSEVHQTFE--GDA   131
dfrA12   SHAIALA----------SELGNELYVAGGAEIYTLALPH--AHGVFLSEVHQTFE--GDA   131
dfrA22   SQAIAIA----------AEHGKELYVAGGAEVYALALPR--ADGVFLSEVHQTFE--GDA   131
dfrA13   SHVSPST----------AEHGKELYVARGAEVYALALPH--ANGVFLSEVHQTFE--GDA   131
dfrA21   SQAIAIA----------AEHGKELYVAGGAEVYALALPH--ANGVFLSEVHQTFE--GDA   131
dfrA26   EEAISLAQSIAL-----IEAVDEIMVLGGGEIYTQALPQ--ADILYLTEVHASVD--GDA   147
dfrA3    DAAL------AL-----LTDCEEAMIIGGGQLYAEALPR--ADRLYLTYIDAQLN--GDT   125
EcDHFR   DEAI------AA-----CGDVPEIMVIGGGRVYEQFLPK--AQKLYLTHIDAEVE--GDT   123

dfrA18   FIPVDPIQNNFHIDSSASE--TVEATV---DETQERIHFATYVRNNQ-------------   189
dfrA19   FIPVDPIQNNFHIDSSASE--TVEATV---DETQERIHFATYVRNNQ-------------   189
dfrA23   TLPMSYIDSKFAIGQGS-I--TIQTTV---DGETHPVKFITYERVQP-------------   186
dfrA10   YVPVELMNKLYSDFKYPENILWVGDPIDS----VYSLSIDKFVRPASLVGVPNDINT---   187
dfrA24   GLDTYI--EDFLSLR--------GTSTPK--------------RKSNLVLPPTPTTP---   185
dfrA8    YVDLAEMVKDYEQNG--------MEEHDL----HTYFTYR---KKELTE-----------   169
dfrA6    FFPSV-P-QSFKQTF--------EQSFSS----NIDYTYQIWTKG---------------   157
dfrA31   FFPSV-P-QSFKQTF--------EQSFSS----NIDYTYQIWAKG---------------   157
dfrA7    NFPKI-P-ENFNLVF--------EQFFLS----NIKYTYQIWKKG---------------   157
dfrA17   KFPIM-P-ENFNLVF--------EQFFMS----NIKYTYQIWKKG---------------   157
dfrA32   KFPIL-P-EGFNLVF--------EQFFVS----NIKYTYQIWKKG---------------   157
dfrA28   YFPES-P-AILGQ-F--------YPRLRS----NIKYSYQIWQRVQCSTIRNCPLCTKRQ   166
dfrA1    YFPEI-P-SNFRPVF--------TQDFAS----NIKYSYQIWQKG---------------   157
dfrA11   YFPEI-P-SNFRPVF--------TQDFAS----NIKYSYQIWQKG---------------   157
dfrA15   YFPEI-P-SSFRPVF--------SQDFVS----NIKYSYQIWQKG---------------   157
dfrA27   FFPEI-P-KKFNVVF--------EQEFHS----NIKYRYQIWQRG---------------   157
dfrA25   FFPNI-P-GKFDVVF--------EQQFTS----NIKYCYQIWQKG---------------   152
dfrA29   FFPNI-P-GKFDVVF--------EQQFTS----NIKYCYQIWQKG---------------   152
dfrA14   FFPSI-P-NTFEVVF--------EQHFTS----NIKYCYQIWKKG---------------   160
dfrA16   FFPSI-P-NTFEVVF--------EQHFTS----NIKYCYQIWKKG---------------   160
dfrA5    FFPNI-P-NTFEVVF--------EQHFSS----NIKYCYQIWQKG---------------   157
dfrA30   FFPNI-P-NFFEVVF--------EQHFSS----NIKYCYQIWKKG---------------   157
dfrA20   FFPFVNW-GEWEEED--------TLEQDKDEKHLYSFNIKKFTR----------------   169
dfrA9    ELDLSLF-KSWKLVS--------EVPTITEN--KTKLIFQIWINPNPI--SEEPTC----   177
dfrA2    FFPMLNE-TEFELVS--------TEFIQA----VIPYTHSVYARRNG-------------   165
dfrA12   FFPMLNE-TEFELVS--------TETTQA----VIPYTHSVYARRNG-------------   165
dfrA22   FFPVLDE-AEFEVVS--------AETVQA----TITYTHSVYARRNG-------------   165
```

```
dfrA13   FFPVLNA-AEFEVVS--------SETIQG----TITYTHSVYARRNG-------------   165
dfrA21   FFPVLNA-AEFEVVS--------SETIQG----TITYTHSVYARRNG-------------   165
dfrA26   FFPDVDL-SQYQETQ--------RQDFEPSGGNPYPFSFVVYQRT---------------   183
dfrA3    HFPDYLS-LGWQELE--------RSTHPADDKNSYACEFVTLSRQR--------------   162
EcDHFR   HFPDYEP-DDWESVF--------SEFHDADAQNSHSYCFKILERR---------------   159

dfrA18   ------   189
dfrA19   ------   189
dfrA23   ------   186
dfrA10   ------   187
dfrA24   ------   185
dfrA8    ------   169
dfrA6    ------   157
dfrA31   ------   157
dfrA7    ------   157
dfrA17   ------   157
dfrA32   ------   157
dfrA28   VASLAG   172
dfrA1    ------   157
dfrA11   ------   157
dfrA15   ------   157
dfrA27   ------   157
dfrA25   ------   152
dfrA29   ------   152
dfrA14   ------   160
dfrA16   ------   160
dfrA5    ------   157
dfrA30   ------   157
dfrA20   ------   169
dfrA9    ------   177
dfrA2    ------   165
dfrA12   ------   165
dfrA22   ------   165
dfrA13   ------   165
dfrA21   ------   165
dfrA26   ------   183
dfrA3    ------   162
EcDHFR   ------   159
```

b

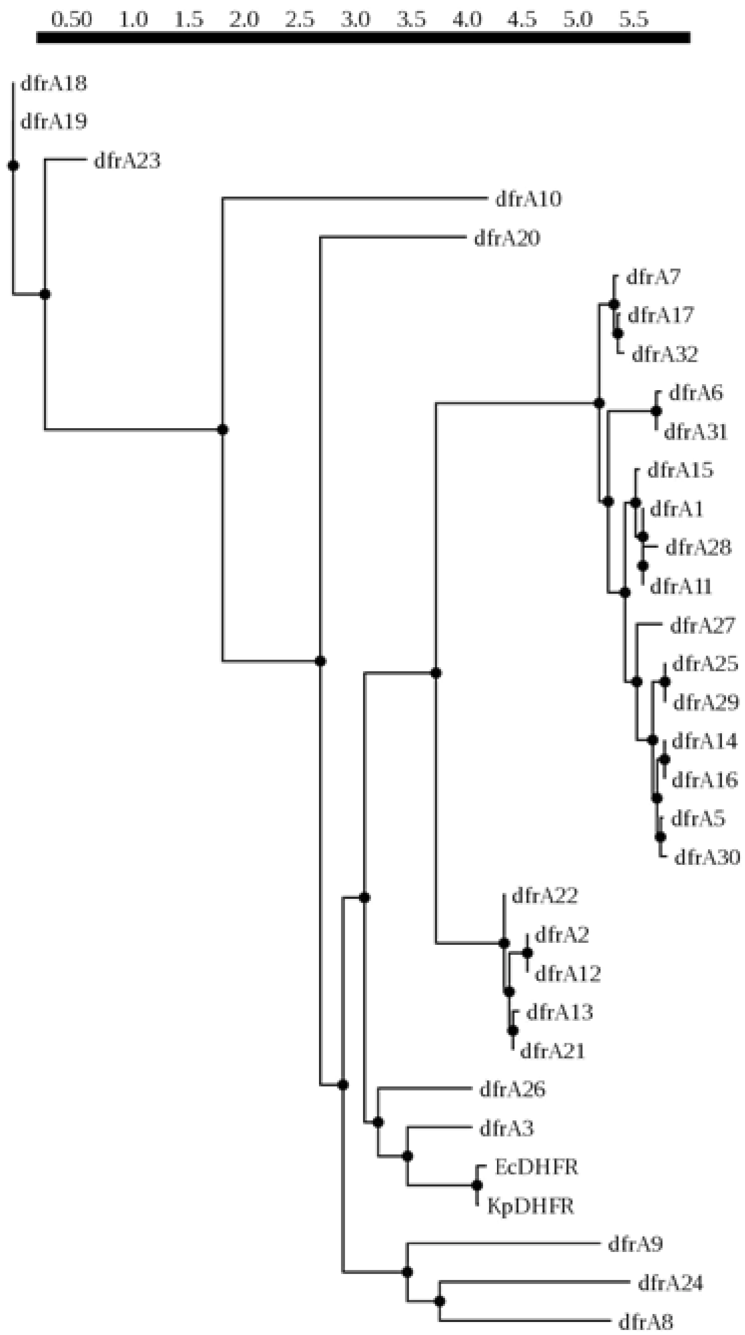

Supplementary Figure S1. Multiple sequence alignment of amino acid sequences by MUSCLE and a phylogenic tree built off the standard Likelihood Ratio Test for all DfrA isoforms.
a A sequence alignment of all known DfrA enzymes and EcDHFR. Residues that interact with trimethoprim are boxed. **b** *K. pneumoniae* DHFR (KpDHFR) was used as a positive control in building the phylogenic tree given the close relation to EcDHFR. Phylogenic analysis was based on the sequence alignment.

E. coli DHFR Mutant T_i (°C)			
	T_i 1	T_i 2	T_i 3
WT	50.6 ± 1.7	58.7 ± 0.5	62.3 ± 2.5
D27E	51.9 ± 1.7	62.1 ± 1.3	-
L28Q	52.0 ± 1.2	57.7 ± 0.8	63.9 ± 1.2
D27E/ L28Q	55.8 ± 0.8	62.0 ± 0.6	66.1 ± 2.1

Supplementary Table S1. Thermal unfolding data of EcDHFR wild type and its mutants.
Data is shown as mean ± standard deviation for n = 2 independent replicates.

a

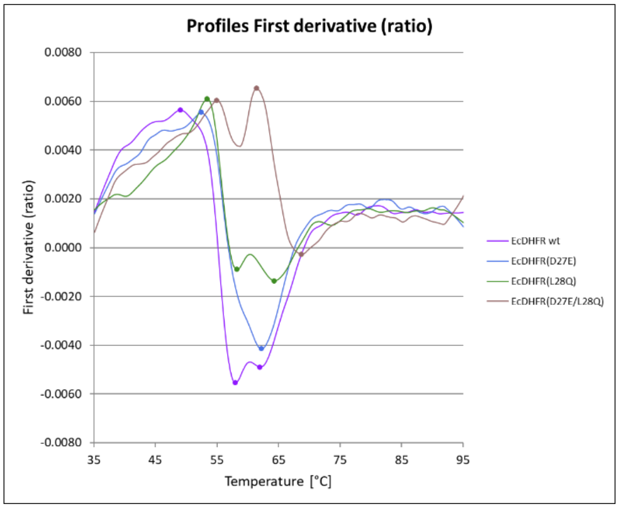

Supplementary Figure S2. Thermal stability of dihydrofolate reductases measured with label-free differential scanning fluorimetry. The first derivative of the fluorescence ratio (Δ*F*350nm/Δ*F*330nm) as a function of temperature are shown in the diagrams. Colored points represent T_i (inflection temperature at which an unfolding transition occurs). Maxima and minima indicate a transition point along the melting curve. Experiments were performed with two independent samples and one representative data for each sample is displayed. **a** Thermal unfolding profile of the wild-type E. coli DHFR (*Ec*DHFR) (brown), *Ec*DHFR(D27E) (blue) and *Ec*DHFR(L28Q) (green) single mutants and EcDHFR (D27E/L28Q) (purple) double mutant.

a

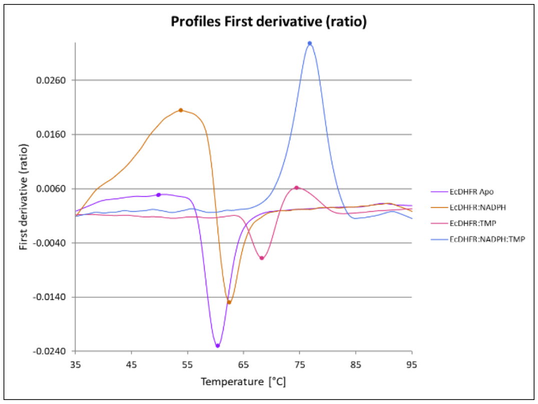

b

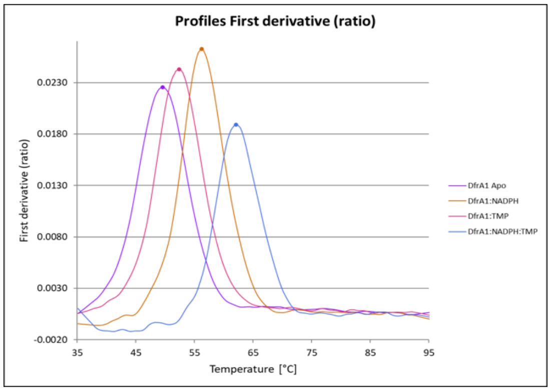

c

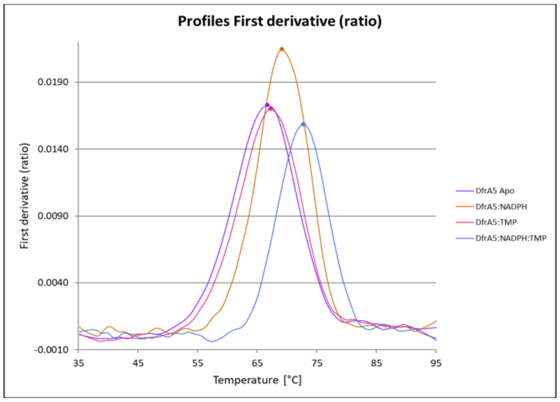

Supplementary Figure S3. Thermal stability profile of wild-type E. coli DHFR (EcDHFR), DfrA1 and DfrA5 upon binding to TMP in presence and absence of co-factor. Representative first derivative plots are shown for fluorescence ratio (350/330 nm) from which Ti values corresponding to the positive and negative peaks in the unfolding profile were determined (n=3). **a** Thermal shift first-derivative curves of EcDHFR Apo (purple), EcDHFR:NADPH (orange), EcDHFR:TMP (red), EcDHFR:TMP:NADPH (blue). **b** Thermal shift first-derivative curves of DfrA1 Apo (purple), DfrA1:NADPH (orange), DfrA1:TMP (red), DfrA1:TMP:NADPH (blue). **c** Thermal shift first-derivative curves of DfrA5 Apo (purple), DfrA5:NADPH (orange), DfrA5:TMP (red), DfrA5:TMP:NADPH (blue).

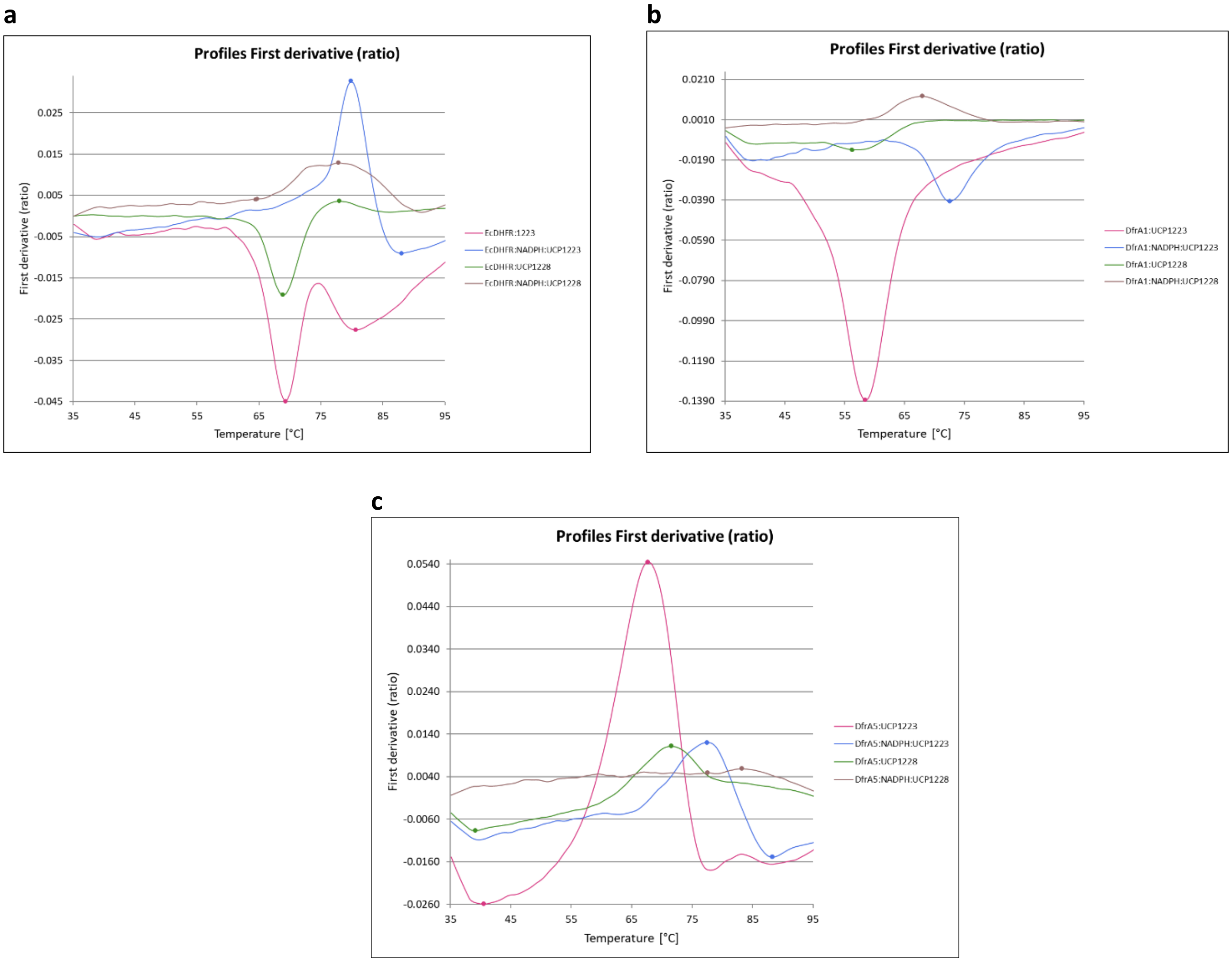

Supplementary Figure S4. Thermal stability profile of wild-type E. coli DHFR (EcDHFR), DfrA1 and DfrA5 upon binding to UCP1223 and UCP1228 in presence and absence of co-factor. Equivalent analysis for all three systems in the presence of PLAs as indicated (n=3). **a** Thermal shift first-derivative curves of EcDHFR:UCP1223 (red), EcDHFR:NADPH:UCP1223 (blue), EcDHFR:UCP1228 (green), EcDHFR:NADPH:UCP1228 (purple). **b** Thermal shift first-derivative curves of DfrA1:UCP1223 (red), DfrA1:NADPH:UCP1223 (blue), DfrA1:UCP1228 (green), DfrA1:NADPH:UCP1228 (purple). **c** Thermal shift first-derivative curves of DfrA5:UCP1223 (red), DfrA5:NADPH:UCP1223 (blue), DfrA5:UCP1228 (green), DfrA5:NADPH:UCP1228 (purple).

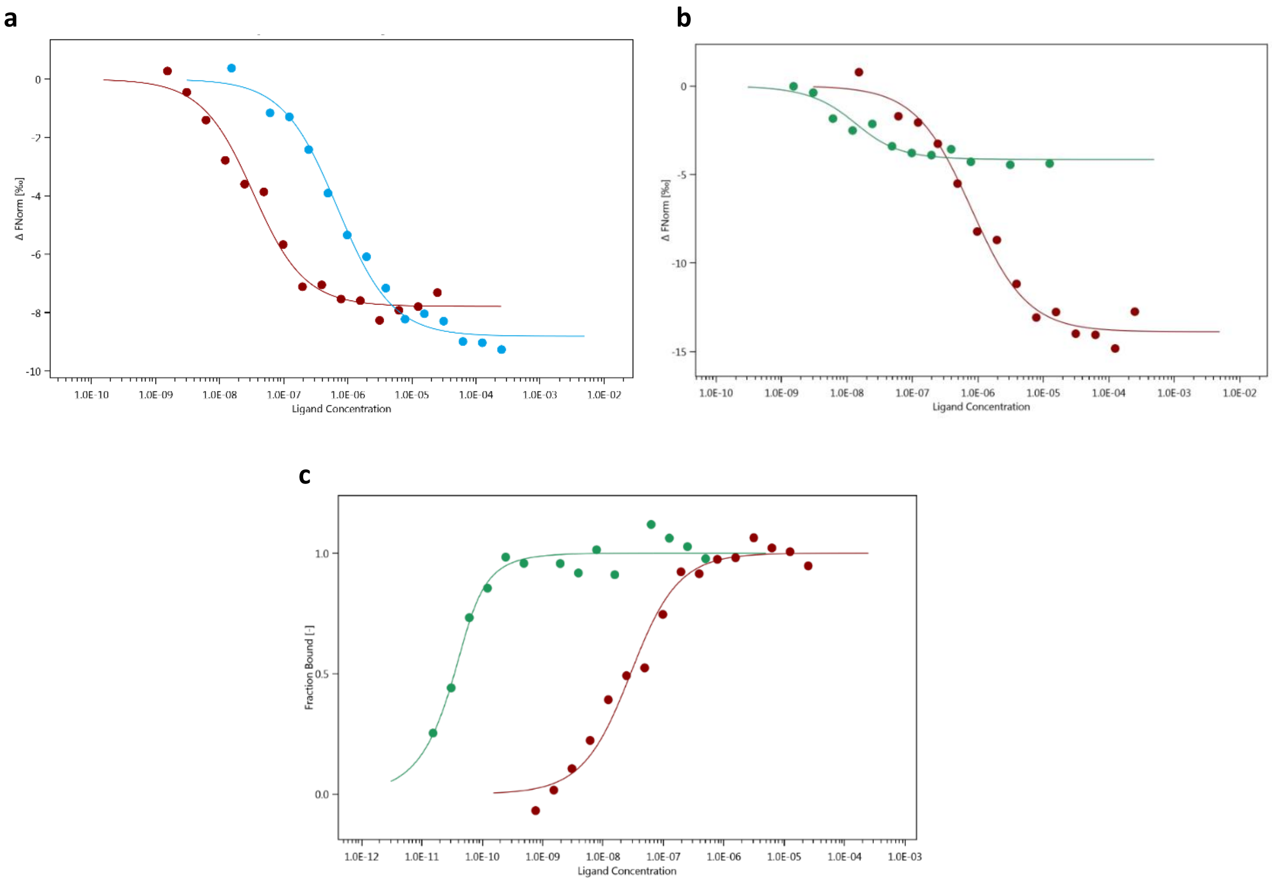

Supplementary Figure S5. Thermophoretic analysis of *Ec*DHFR interactions with NADPH and TMP by MST. Normalized binding response (ΔF_{norm} of fraction bound) of the increasing concentrations of the non-fluorescent ligand to the constant concentration of the red-NTA labeled enzyme. The experiments were performed using medium MST power and between 40 to 80% LED power, at 23 °C. The MST traces were recorded with the following parameters: 5 s MST power of, 30 s MST power on and 5 s MST power of. Data collection was performed with MO.Control 2 (Nano-temper Technology GmbH). The reported measurements are the combination of the fast, local environment dependent responses of the fluorophore to the temperature jump and the slower diffusive thermophoretic fluorescence changes for the enzyme, pre and post incubation step. Equilibrium binding curves are depicted as thick lines and dots are representing up to 16 concentration sampling schemes, expressed as individual data points. The Data were fitted to a single-site binding model accounting for ligand depletion. The Kd values obtained from the best fit are reported in Table 4. **a** Titration of 50 nM red-NTA labeled *Ec*DHFR apo form into a serial dilution of NADPH (blue) and TMP (red) ranging from 250 µM to 0.015 µM and 25 µM to 0.000763 µM respectively. Fit of the changes in the thermophoresis signal yielded a Kd of 661 nM ± 98 nM for NADPH and 24.5 nM ± 8.2 nM for TMP. **b** Binding of NADPH to red-NTA labeled *Ec*DHFR with and without 1 µM TMP preincubation step. Shift in thermophoretic movement after titration of NADPH from 250 µM to 0.015 µM against 50 nM *Ec*DHFR apo form resulted in Kd of 771 ± 145 nM (red). Titration of NADPH from 12.5 µM to 0.00153 µM against 10 nM of the enzyme preincubated with TMP caused a less pronounced response in MST signal, yielding Kd of 8.1 ± 4.0 nM (green). **c** Binding of TMP to red-NTA labeled *Ec*DHFR in presence and absence of co-factor. Titration of TMP from 25 µM to 0.0007 µM against 50 nM apo enzyme yielded Kd of 23.57 nM ± 5.15 nM (red), while addition of TMP from 0.5 µM to 0.000015 µM to 10 nM labeled, pre-formed binary complex with NADPH gave Kd of 0.011nM ± 0.0075 nM (green). A high margin of error for the ternary binding events is due to a limitation of the instrument to accurately measure protein-ligand interactions in a low picomolar range.

a b

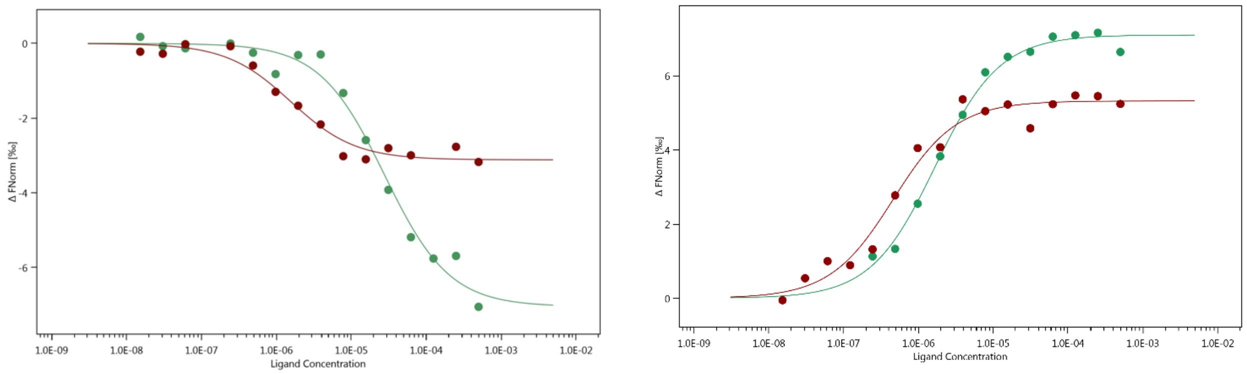

Supplementary Figure S6. Thermophoretic analysis of DfrA1 interactions with NADPH and TMP by MST. Equivalent analysis for DfrA1 as indicated. **a** Binding of NADPH to red-NTA labeled DfrA1 apo form and to preformed DfrA1:TMP complex. Titration of NADPH from 500 µM to 0.0244 µM against 50 nM DfrA1 apo form resulted in Kd of 1.65 ± 0.23 µM (green). Binding of NADPH from 500 µM to 0.015 µM to 50 nM DfrA1 preincubated with 100 µM TMP yielded Kd of 0.43 ± 0.09 µM (red). **b** Binding of TMP to red-NTA labeled DfrA1 as a binary (DfrA1:TMP) and a ternary (DfrA1: NADPH:TMP) complex. Titration of TMP from 500 µM to 0.015 µM with 50 nM DfrA1 apo from gave Kd of 27.79 µM ± 4.87 µM (green), while addition of TMP from 500 µM to 0.015 µM against the same enzyme preincubated with 100 µM NADPH gave Kd of 1.53 µM ± 0.36 µM (red).

a b

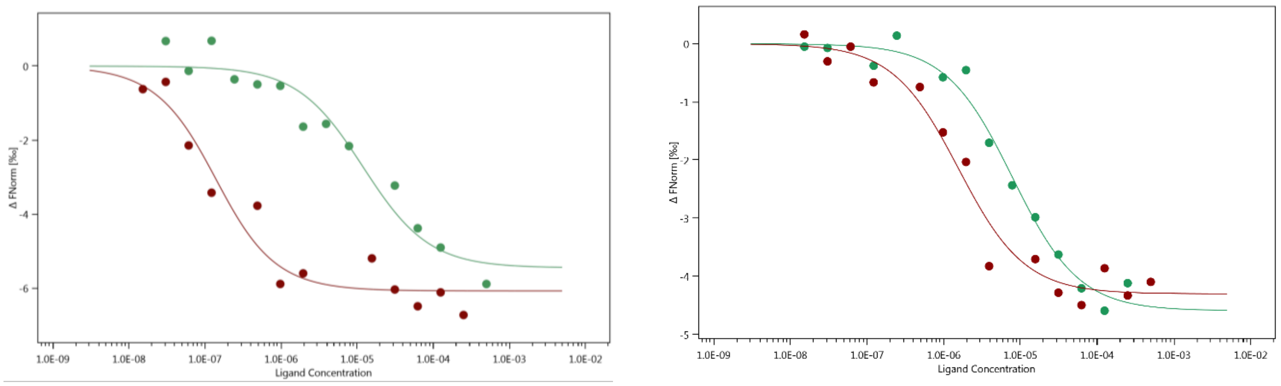

Supplementary Figure S7. Thermophoretic analysis of DfrA5 interactions with NADPH and TMP. Equivalent analysis for DfrA5 as indicated. **a** Binding of NADPH to red-NTA labeled DfrA5 apo form and to preformed DfrA5:TMP complex. Titration of NADPH from 500 µM to 0.015 µM against 50 nM labeled DfrA5 apo form yielded Kd of 7.47 ± 1.66 µM (green), while titration of NADPH against 50 nM labeled DfrA5 preincubated with 50 µM TMP resulted in Kd equal 1.54 µM ± 0.38 µM (red). **b** Binding of TMP to red-NTA labeled DfrA5 apo form and to preformed DfrA5:NADPH complex. Titration of TMP from 500 µM to 0.015 µM against 50 nM labeled DfrA5 apo from yielded Kd of 11.93 µM ± 4.45 µM (green), while titration of TMP from 250 µM to 0.015 µM against 50 nM preformed complex of DfrA5 with 100 µM NADPH derived a Kd value of 0.113 µM ± 0.056 µM (red).

a

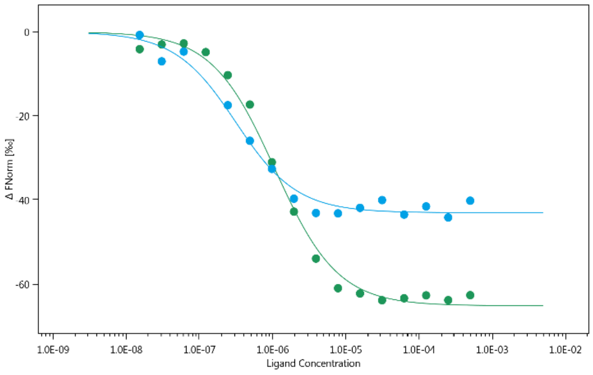

b

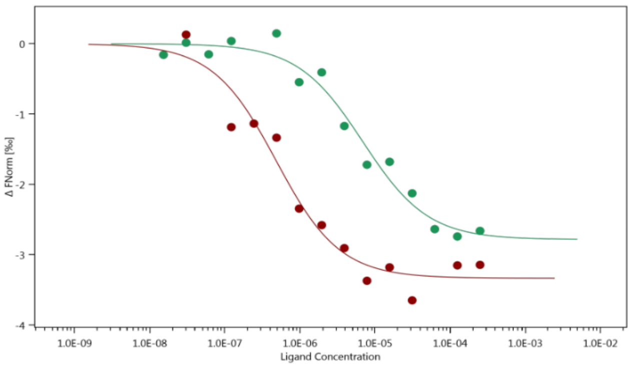

Supplementary Figure S8. MST binding response of NADPH to preformed complexes of DfrA1 and DfrA5 with 100 µM UCP1228 respectively. By fitting the change in the thermophoretic movement upon titration of NADPH to a constant amount of the labeled enzyme alone and in the presence of UCP1228, a different binding constant of Kd was determined. **a** Binding of NADPH to red-NTA labeled DfrA1 in presence and absence of UCP1228. Titration of NADPH from 500 µM to 0.015 µM against 50 nM labeled DfrA1 apo form yielded Kd of 1.04 ± 0.11 µM (green), while titration of co-factor into 50 nM labeled protein preincubated with 100 µM UCP1228 complex resulted in Kd equal to 0.29 µM ± 0.05 µM (blue). **b** Binding of NADPH to red-NTA labeled DfrA5 apo form and to preformed DfrA5:UCP1228 complex. Titration of NADPH from 250 µM to 0.015 µM against 50 nM labeled DfrA5 apo from yielded Kd of 6.84 µM ± 1.60 µM (green), while titration of NADPH from 250 µM to 0.031 µM against 50 nM labeled DfrA5 preincubated with 100 µM UCP1228 derived a Kd value of 0.45 µM ± 0.15 µM (red).

Protein	Inhibitor	Co-factor observed	Resolution (Å)	PDB ID
E. coli DHFR	Trimethoprim	-	2.4	7NAE
E. coli DHFR	Trimethoprim	+	3.0	7MYM
E. coli DHFR	UCP1223	-	2.1	7REB
E. coli DHFR	UCP1228	-	1.9	7MQP
DfrA1	Trimethoprim	-	2.2	7MYL
DfrA1	UCP1223	+	1.4	7RGJ
DfrA1	UCP1228	+	1.8	7REG
DfrA5	Trimethoprim	+	2.6	7R6G
DfrA5	UCP1223	+	2.2	7RGK
DfrA5	UCP1228	+	1.9	7RGO

Supplementary Table S2. Summary of crystal structures described in this study

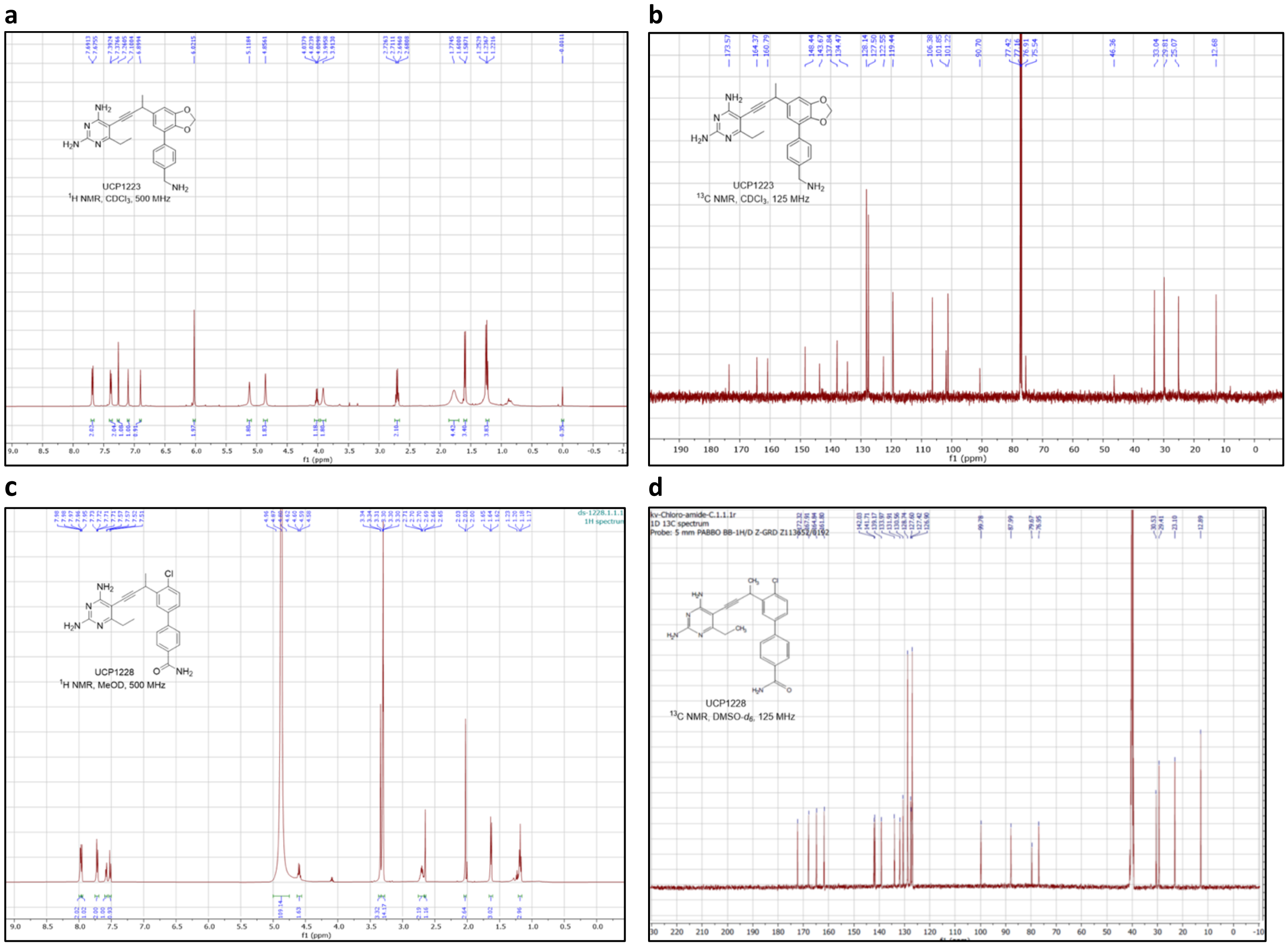

Supplementary Figure S9. Characterization of UCP1223 and UCP1228 by ^{1}H and ^{13}C nuclear magnetic resonance (NMR). **a** ^{1}H and **b** ^{13}C NMR spectra of UCP1223 in $CDCl_3$. **c** ^{1}H and **d** ^{13}C NMR spectra of UCP1228 in MeOD and DMSO-d_6 respectively.

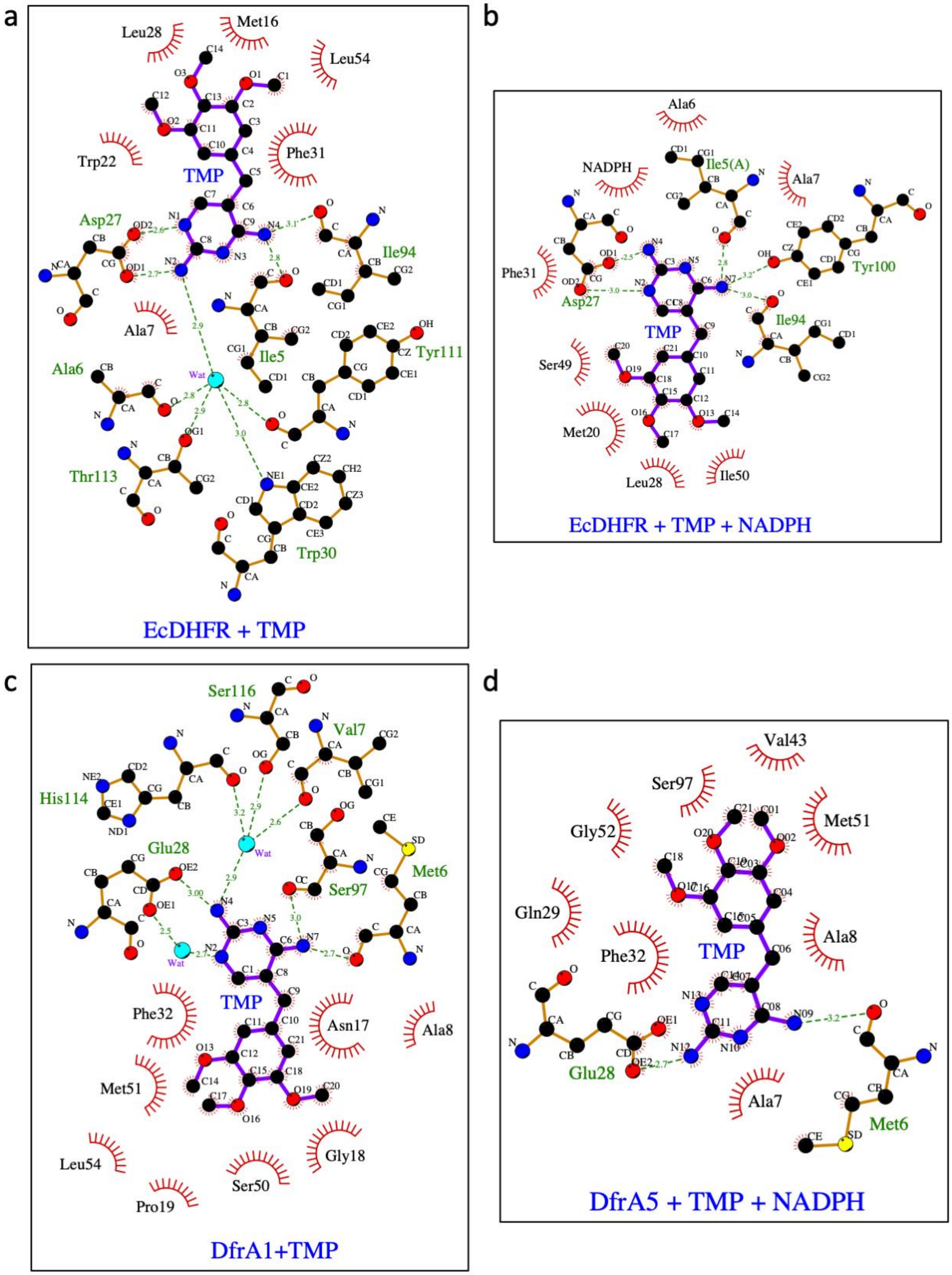

Supplementary Figure S10. Ligand-protein interaction diagrams for TMP-protein interactions from the crystal structures presented in this study. The complexes are**:** **a** EcDHFR:TMP (PDB entry 7NAE), **b** EcDHFR:TMP:NADPH (7MYM), **c** DfrA1:TMP (7MYL), **d** DfrA5:TMP:NADPH (7R6G). Residues involved in van der Waals contacts are depicted as red spoked arcs. Hydrogen bonds are shown as green dotted lines. Only conserved water molecules are included and are shown in cyan.

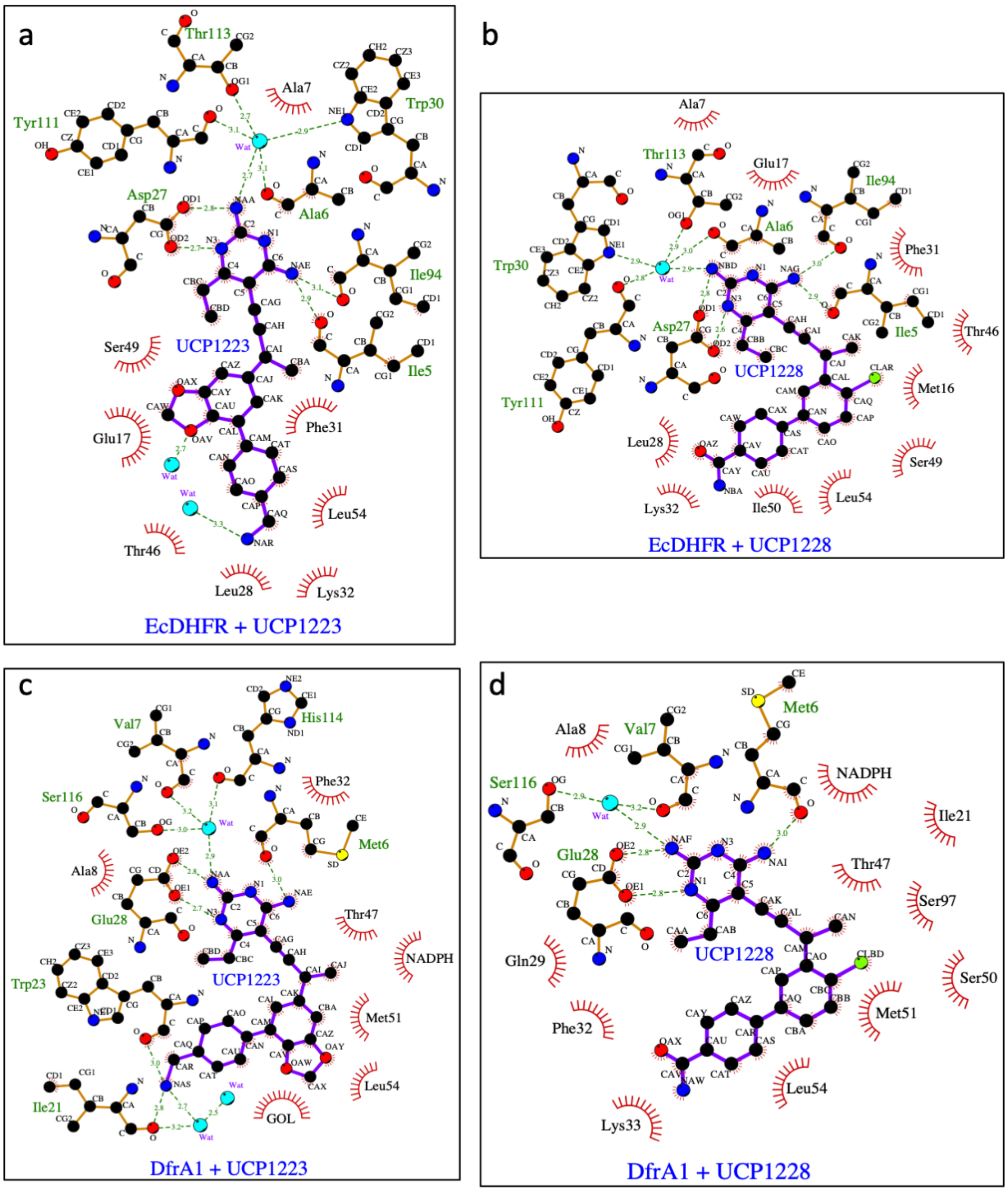

Supplementary Figure S11. Ligand-protein interaction diagrams for UCP1223 and UCP1228-protein interactions from the crystal structures presented in this study. The complexes are: **a** EcDHFR:UCP1223 (PDB entry 7REB), **b** EcDHFR:UCP1228:NADPH (7MQP), **c** DfrA1:UCP1223:NADPH (7RGJ), **d** DfrA1:UCP1228:NADPH (7REG). Residues involved in van der Waals contacts are depicted as red spoked arcs. Hydrogen bonds are shown as green dotted lines. Only conserved water molecules are included and are shown in cyan.

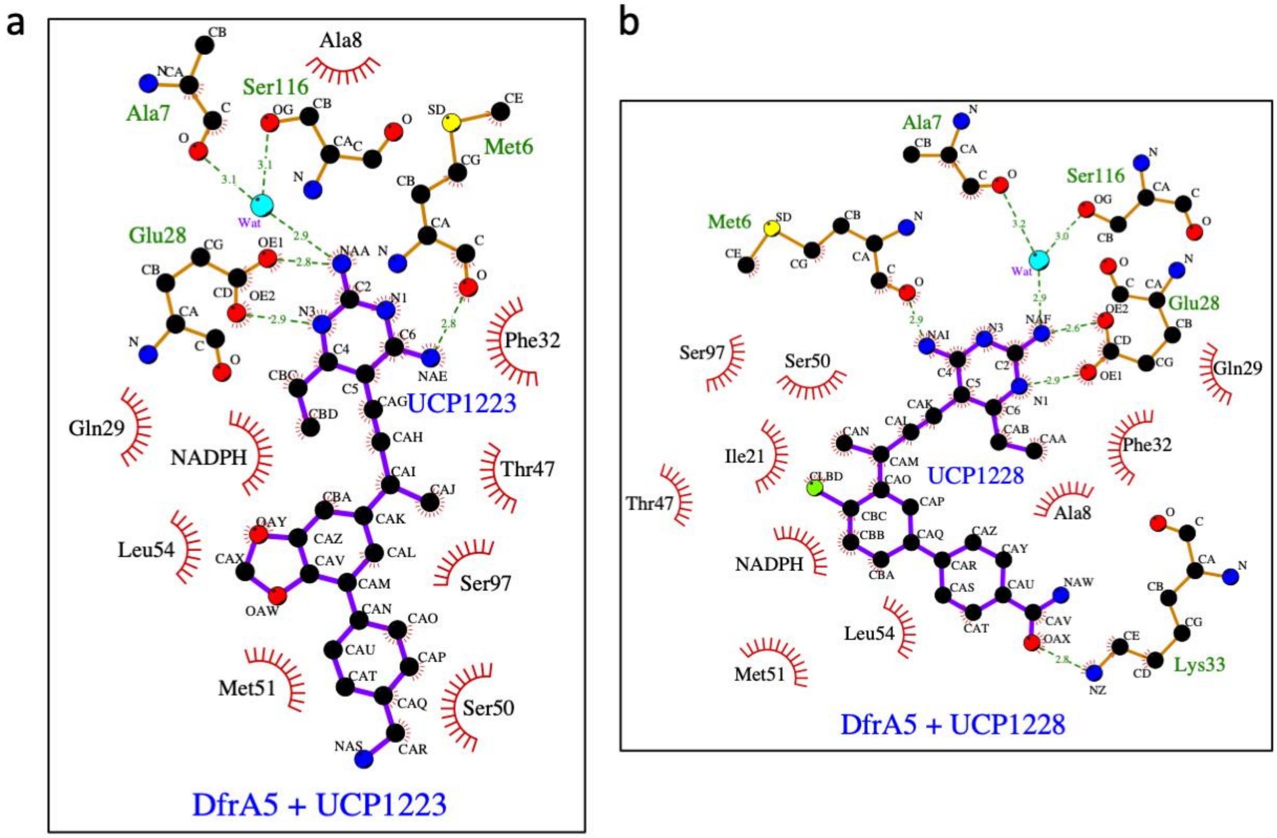

Supplementary Figure S12. Ligand-protein interaction diagrams for UCP1223 and UCP1228-protein interactions from the crystal structures of DfrA5 presented in this study. The complexes are**: a** DfrA5:UCP1223:NADPH (PDB entry 7RGK) **b** DfrA5:UCP1228:NADPH (7RGO). Residues involved in van der Waals contacts are depicted as red spoked arcs. Hydrogen bonds are shown as green dotted lines. Only conserved water molecules are included and are shown in cyan.

a

Compound ID	MIC (μg/mL)[a]			MIC (μg/mL)[a]		
	BW25113 w/o SMX	JW0451 w/o SMX	Fold difference	BW25113 w/o SMX	BW25113 w/ SMX	Fold difference
**TMP**	0.312	0.04	8	0.312	0.078	4
**UCP1223**	20	0.625	32	20	0.625	32
**UCP1228**	10	1.25	8	10	0.625	16

b

Compound ID	MIC (μg/mL)[a]			MIC (μg/mL)[a]		
	*Ec*DHFR[b] w/o SMX	DfrA1[c] w/o SMX	DfrA5[d] w/o SMX	*Ec*DHFR[b] w/ SMX	DfrA1[c] w/ SMX	DfrA5[d] w/ SMX
**TMP**	0.625	>20	>20	0.078	>20	>20
**UCP1223**	20	>20	>20	1.25	20	10
**UCP1228**	20	>20	>20	1.25	20	20

Supplementary Table S3. Comparative antimicrobial activities of TMP, UCP1223 and UCP1228 alone and paired with SMX (1:19) a against *E. coli* BW25113 ATTC strain and *E. coli JW0451* acrB mutant strain, **b** *E. coli* BL21(DE3) strain overexpressing *Ec*DHFR wt, DfrA1 and DfrA5 respectively. [a]The Minimum Inhibitory Concentration (MIC) values represent 80% inhibition of bacterial growth after 18 to 20 hours of incubation at 37°C from duplicates. [b]BL21(DE3) strain transformed with pET41a-*Ec*DHFR wt plasmid, [c]BL21(DE3) strain transformed with pET41a-DfrA1 plasmid and [d]BL21(DE3) strain transformed with pET24a-DfrA5 plasmid.
